# Is Hypovitaminosis D Related to Incidence of Type 2 Diabetes and High Fasting Glucose Level in Healthy Subjects: A Systematic Review and Meta-Analysis of Observational Studies

**DOI:** 10.3390/nu10010059

**Published:** 2018-01-10

**Authors:** Shamaila Rafiq, Per Bendix Jeppesen

**Affiliations:** Department of Clinical medicine, Aarhus University Hospital , Aarhus University, Tage-Hansen’s Gade 2, 8000 Aarhus C, Denmark; shamailarafiq@clin.au.dk

**Keywords:** 25 (OH) vitamin D, Vitamin D, Type 2 diabetes, hyperglycemia, meta-analysis

## Abstract

There is evidence that vitamin D status is associated with type 2 diabetes. Many observational studies have been performed investigating the relationship of vitamin D status and circulating biomarkers of glycemic regulation. To find out whether this association holds, we conducted a systematic review and meta-analysis of cross sectional and longitudinal studies. We searched Pubmed, Medline and Embase, all through June 2017. The studies were selected to determine the effect of vitamin D on the parameters of glucose metabolism in diabetic and non-diabetic subjects. Correlation coefficients from all studies were pooled in a random effects meta-analysis. The risk of bias was assessed using Grading of Recommendations Assessment, Development and Evaluation (GRADE) system. We found significant inverse relationship of vitamin D status with glycemic level in both diabetic (*r* = −0.223, 95% CI = −0.184 to −0.261, *p* = 0.000) and non-diabetic (*r* = −0.073, 95% CI = −0.052 to −0.093, *p* = 0.000) subjects. This meta-analysis concludes that hypovitaminosis D is associated with increased risk of hyperglycemia both in diabetic and non-diabetic subjects. A future strategy for the prevention of impaired glycemic regulation could be individualized supplementation of vitamin D.

## 1. Introduction

Type 2 diabetes is among the most common diseases worldwide, which affects more than 422 million individuals globally; this number has been increasing at a rate of 9.2 million annually since 1980 (World Health Organization report, 2016). There is an urgent need to reduce these numbers and the implementation of early preventive measures provides a good possibility to decrease the prevalence of type 2 diabetes [1]. There are still no promising treatments for those who develop advanced type 2 diabetes. It is suggested that type 2 diabetes is related to modifiable risk factors, thus, by addressing the risk factors through implementation of preventive measures, the risk of type 2 diabetes could be reduced [2]. It is very important to identify these risk factors to rescue those who are at greater risk of the disease. Multifunctional vitamin D is required for the skeletal and extra-skeletal functions in the human body [3,4]. The functions of this vitamin in skeleton are very well understood. There is, however, evidence that the hypovitaminosis D also plays a role in the development of diseases such as autoimmune diseases, cancers and type 2 diabetes [3,4]. The presence of vitamin D receptors on the beta cells, adipose tissues and skeletal muscle cells [5,6,7] indicates the function of this vitamin in the glucose metabolism. Vitamin D can enhance the synthesis of insulin hormone and its release from the beta cells; hence, it plays a role in glucose metabolism. It can also increase the expression of insulin receptor and suppress the pro-inflammatory cytokines that may promote insulin resistance [8].

There is a concurrent increase in the prevalence of type 2 diabetes and hypovitaminosis D worldwide [2], which is also indicative of an effect of vitamin D deficiency against the onset of type 2 diabetes. Vitamin D deficiency, which may be a key factor for Type 2 diabetes development [9,10,11], is prevalent in over one billion individuals in the world [12]. Hyperglycemia is characteristic of type 2 diabetes. Vitamin D has been shown to exert a compensatory effect in the correction of hyperglycemia. Hypovitaminosis D might have an effect on insulin secretion. Possibly, the increased intracellular calcium dephosphorylates glycogen synthase and impairs glucose transporter (GLUT-4). Hypovitaminosis D is known to increase the secretion of parathyroid hormone which results in elevated levels of intracellular calcium. This perpetually increased calcium concentration inhibits the cells from sensing calcium fluxes essential for insulin related actions. These calcium fluxes are also necessary for the beta cells to secrete insulin [7].

Vitamin D as a vitamin is unique as it can be synthesized in the skin in addition to its dietary sources. Dietary sources contain an insufficient quantity of vitamin D unless fortified with this vitamin. It is necessary, therefore, to have sun exposure to get maximum vitamin D for those who are deficient in vitamin D. This cutaneous synthesis of vitamin D, however, is regulated by the excess sunlight exposure which photo-degrade the extra vitamin D to avoid toxicity [8]. Whether the deficiency of vitamin D is related with the onset of type 2 diabetes gains a lot of importance, because this condition highly prevails and because there seems an option for the reversibility of the disease conditions. This systematic review and meta-analysis was performed based on this background, investigating whether the hypovitaminosis D could be the potential cause for the onset of type 2 diabetes.

## 2. Materials and Methods

The articles were searched systematically by literature search of Pubmed, Medline and EMBASE from inception until June 2017 considering key words “25(OH) D”, “Cholecalciferol”, “25-hydroxyvitamin D” or Vitamin D3” in combination with “type 2 diabetes” or “T2D” or “Fasting plasma glucose” or “FPG” or “HBA1C” or “HOMA-IR” or “Homeostasis model assessment of insulin resistance” or “fasting plasma insulin”. Each keyword was searched as free term and in combination with medical subject headings (MeSH)term in Pubmed and thesaurus used in EMBASE database (EMTREE) in EMBASE. The articles were eligible if: (1) vitamin D status was presented as 25(OH) D as it is thought to be the best indicator of vitamin D; (2) included subjects (diabetic and non-diabetic) were older than 18 years with all ranges of BMI; and (3) it was written in English. The studies were excluded if they were done: (1) on animals or cell lines; or (2) on patients with diseases other than type 2 diabetes. In addition, editorials, reviews, case reports and commentaries were excluded. The included articles were hand searched again for additional references.

### Effect Size Measures and Statistical Analysis

After electronically searching the studies, the articles were selected with analysis of title and abstract according to inclusion criteria. The articles were read in full when the abstracts lacked the information. Data obtained from the studies were cross checked. A summary measure based on correlation coefficient was used for the outcome studied. For the studies of immigrants, the host country latitude was considered. One study [13] did not mention the method for vitamin D determination; therefore, we excluded it from the corresponding meta-regression analysis.

The Q test is not considered to be a good test to assess the heterogeneity among studies in meta- analysis [14]; therefore, we used an alternative approach for the assessment of heterogeneity, i.e., *I*^2^. The *I*^2^ is a quantitative analysis of heterogeneity and it is more reliable, as it describes heterogeneity in the form of percentage. Random effect model of meta-analysis was used to pool the data from eligible studies as we collected the studies from a variety of populations all over the world. All statistical analyses were performed using comprehensive meta-analysis software version 3 (Biostat, Inc., Englewood, NJ, USA).

## 3. Results

**Figure 1:**
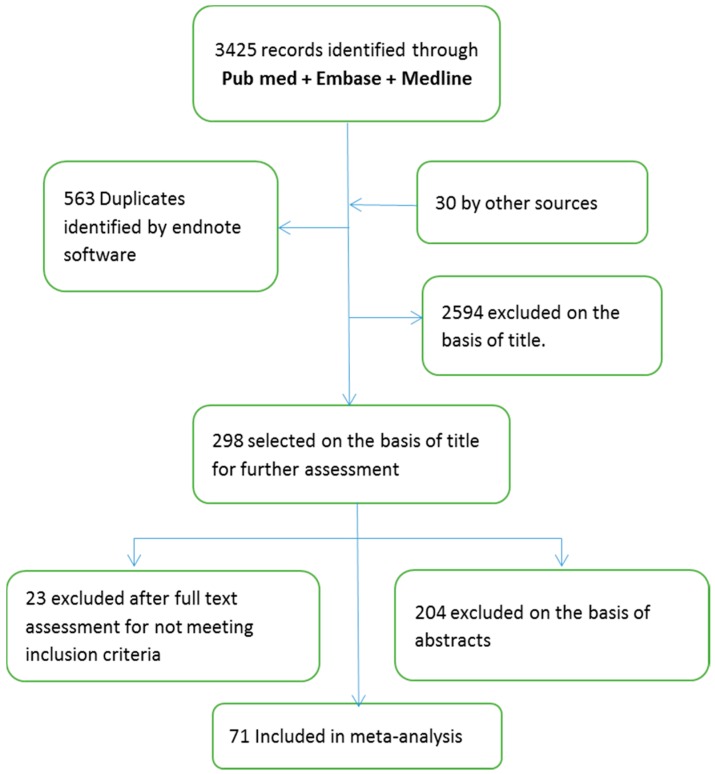
Flow diagram presenting the number of studies screened and assessed for eligibility and final number of references in the included studies.

A total of 3425 observational studies were identified electronically from Pubmed, Embase and Medline. Thirty records were identified by other sources. The references were screened for duplication using endnote software and 563 references were removed. The titles were screened in the first round of selection, where 298 articles were selected for abstract assessment. Out of these 298 articles, 94 were eligible for full text assessment and systematically assessed for inclusion. Twenty-three studies did not meet the inclusion criteria or lacked the sufficient data reporting, thus were excluded. Seventy-one studies met the inclusion criteria and were used for the meta-analysis (Figure 1).

### 3.1. Excluded Studies

Four studies were excluded because their study plan was not in line with our study plan [15,16,17,18,19]. Ten studies [20,21,22,23,24,25,26,27] were excluded because the data were not compatible to calculate correlation coefficient. Five studies [28,29,30,31,32,33] were excluded because the parameters were calculated for mixed population (healthy and type 2 diabetes patients). Two studies were excluded because the number of participants in each vitamin D Quintile had not been mentioned [34,35]. One study [36] was excluded because it was only an abstract from a conference presentation. We excluded all interventional studies with vitamin D supplementation, but the possibility of individual vitamin D intake cannot be ruled out in the included studies.

### 3.2. Included Studies

#### 3.2.1. Meta-Analysis for the Type 2 Diabetes Patient Studies

**Figure 2:**
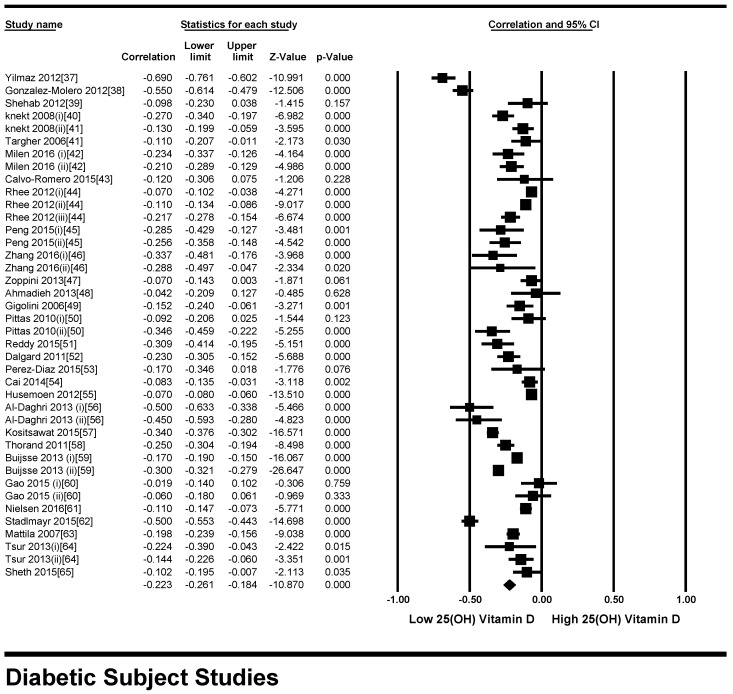
Forest plot showing the effect of vitamin D on type 2 diabetes. Data calculated from the random-effects model are presented as correlations and 95% CI (*I*^2^ = 95.932%, *p* = 0.000).

**Figure 3:**
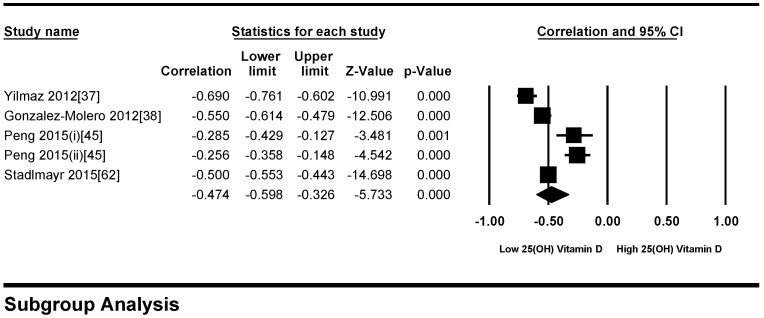
Forest plot showing the effect of vitamin D on type 2 diabetes in studies used ECLIA method for the determination of vitamin D. Data calculated from the random-effects model are presented as correlations and 95% CI (*I*^2^ = 94.757%, *p* = 0.000).

**Figure 4:**
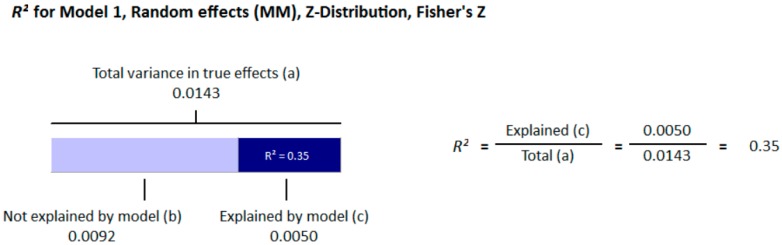
Regression Analysis of diabetic studies for the moderator, “method of determination of vitamin D”: Graphic representation of R-squared for the effect of method of determination of vitamin D on between studies variation in correlation.

**Figure 5:**
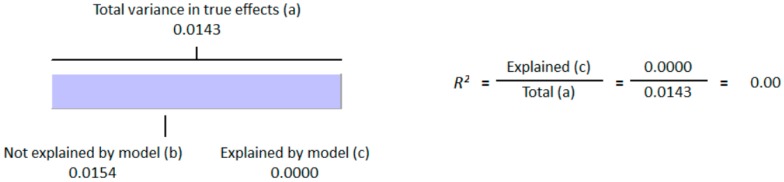
Regression Analysis of diabetic studies for the moderator, “Latitude”: Graphic representation of R-squared for the effect of latitude on between studies variation in correlation.

**Figure 6:**
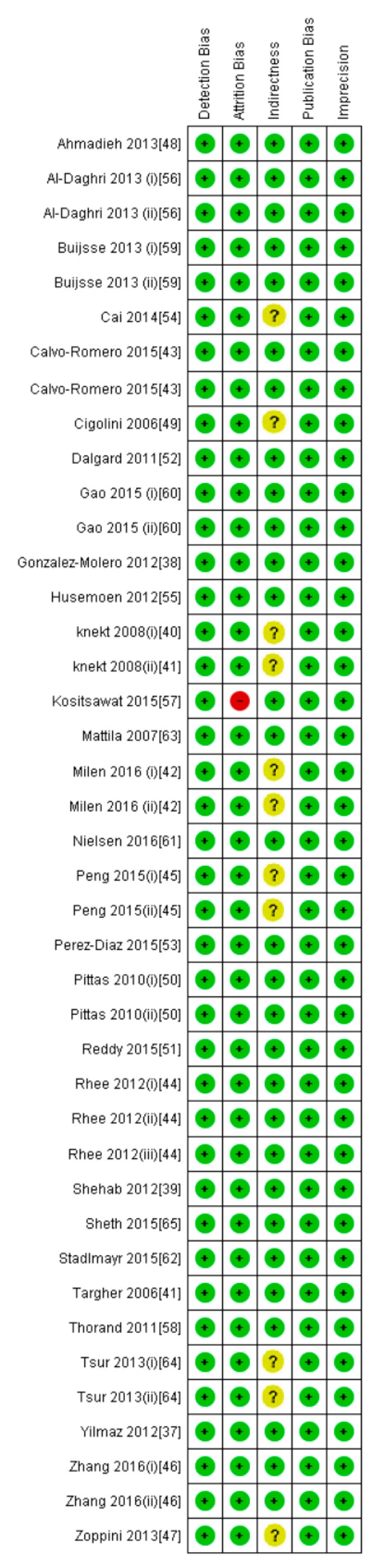
Risk of bias, including the assessment of each item by the authors. Data are shown as percentage for all diabetic patient studies.

**Figure 7:**
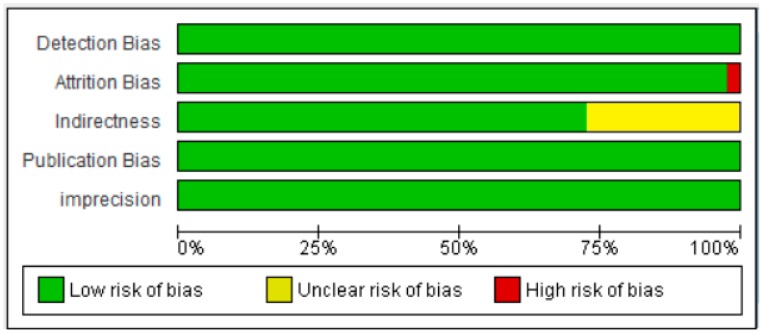
Risk of bias summary for each diabetic patient study as determined by the judgments of the authors.

All participants of the included studies were more than 18 years of age. The study subjects included were from 16 different countries, nine from Europe and America and the other seven from Asia. Nine studies determined serum vitamin D concentrations by radioimmunoassay (RIA), one study by high performance liquid chromatography HPLC, four by enzyme linked immunosorbant assay (ELISA) and five by liquid chromatography mass spectrometery (LC-MS), while ten studies used the chemiluminescence (CLIA) method, out of which four studies used electrochemiluminescence (ECLIA) method. Because of this large variability in the population and selection of methods for the determination of vitamin D, we selected random effect model for meta-analysis. The meta-analysis showed an overall significant inverse relationship (*r* = −0.223, 95% CI = −0.184 to −0.261, *p* = 0.000) of hypovitaminosis D with type 2 diabetes (Figure 2). The correlation for almost all of the studies lies between −0.5 and 0, except Yilmaz 2012 [37], and Gonzalez-Molero 2012 [38], who showed relatively large inverse relationships, *r* = −0.690 and *r* = −0.550, respectively, which could be explained by the method of determination of vitamin D, as both of the studies used electrochemiluminescence (ECLIA) method. The analysis of four articles with vitamin D determination by ECLIA method was performed. The results of these observational studies when isolated showed substantial effects on the correlation between vitamin D status and type 2 diabetes (*r* = −0.474, 95% CI = −0.326 to −0.598, *p* = 0.000) (Figure 3). The *R*^2^ graphics showed that 35% of the heterogeneity was because of the method of determination of vitamin D (*R*^2^ = 0.35, *p* = 0.00) (Figure 4), however no variability in the studies was observed due to latitude (*R*^2^ = 0.00, *p* = 0.00) (Figure 5). All of the studies considered the subjects as diabetic if they had fasting plasma glucose (FPG) level greater than 126 mg/dL or HbA1C more than 6.5%. Figure 6 and Figure 7 show the summary of the GRADE assessments for the included studies in the meta-analysis.

#### 3.2.2. Meta-Analysis for the Non-Diabetic Subject Studies

**Figure 8:**
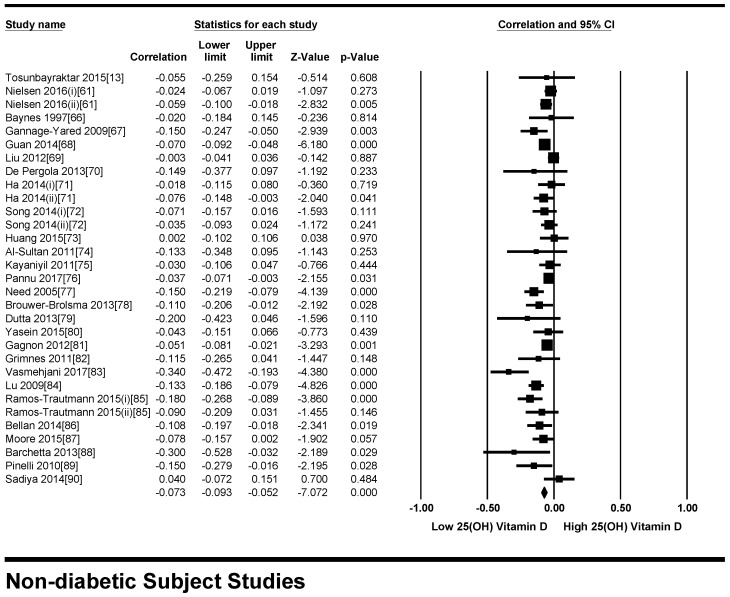
Forest plot showing the effect of vitamin D on fasting plasma glucose in non-diabetic subjects. Data calculated from the random-effects model are presented as correlations and 95% CI (*I*^2^ = 55.463%, *p* = 0.000).

**Figure 9:**
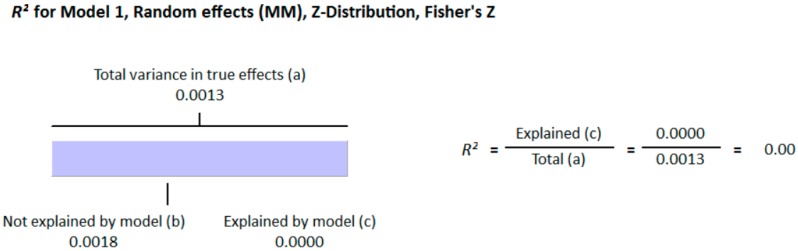
Regression Analysis of non-diabetic studies for the moderator, “method of determination of vitamin D”: Graphic representation of R-squared for the effect of method of determination of vitamin D on between studies variation in correlation.

**Figure 10:**
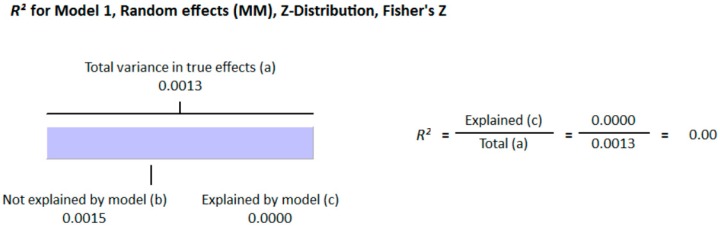
Regression Analysis of non-diabetic studies for the moderator, “Latitude”: Graphic representation of R-squared for the effect of latitude on between studies variation in correlation.

**Figure 11:**
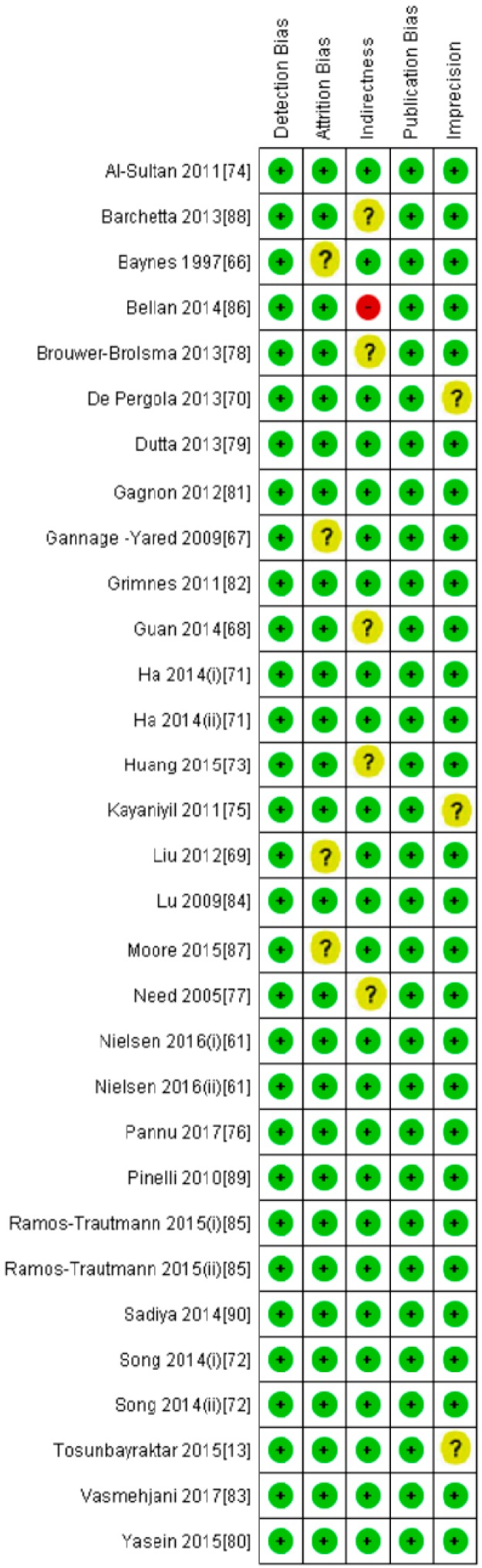
Risk of bias, including the assessment of risk of bias for each item by the authors. Data are shown as percentages for all non-diabetic patient studies.

**Figure 12:**
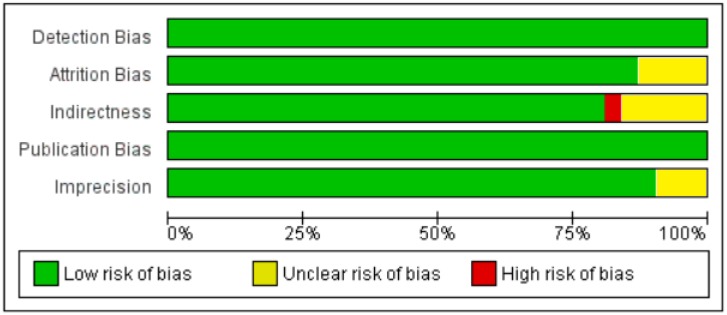
Risk of bias summary for each non-diabetic patient study as determined by the judgments of the authors.

As shown in Figure 8, the serum vitamin D concentration is inversely correlated (*r* = −0.073, 95% CI = −0.052 to −0.093, *p* = 0.000) with fasting plasma glucose levels in non-diabetic subjects. Twenty-eight studies were included in this meta-analysis, conducted in 20 different countries, among those 10 were from continent Asia and Australia and the remaining 10 were from the continent Europe and America. Seven different methods were used to evaluate the concentration of 25(OH) vitamin D. The between study variability as described by the values of tau-squared (Tau-sequared= 0.001, *p* = 0.00) are low (0.01%) (Figure 9 and Figure 10). No moderator (Latitude and method of determination of vitamin D) showed any effect (*R*^2^ = 0.00, *p* = 0.00) on the overall point estimate (Figure 11 and Figure 12). No studies used electrochemiluminescence (ECLIA) method for the determination of vitamin D in this part of meta-analysis. The random effect model was used to pool the data in the meta-analysis. The summary of GRADE assessment for this part of meta-analysis is shown in Figure 11 and Figure 12.

## 4. Discussion

In this meta-analysis, the results of 71 studies both on diabetic and non-diabetic subjects provided substantial evidence of association of vitamin D with type 2 diabetes. The pooled results for correlation coefficient from diabetic patient studies showed a more pronounced relationship of vitamin D with hyperglycemia compared to the non-diabetic subjects. The funnel plots from diabetic subject studies showed no obvious Risk of bias, except for the two studies (37, 38). Moreover, we found that these studies, along with two other studies that (67, 84) showed greater relative correlation, had used ECLIA method for the determination of vitamin D. We did meta-regression for the moderator “method of determination of vitamin D” and found that 35% of heterogeneity was because of this moderator (*R*^2^ = 0.35) (Figure 4). We further conducted the subgroup analysis for these four studies (37, 38, 67, 84) and found that the pooled correlation coefficient had more than doubled (*r* = −0.474) compared to the overall (*r* = −0.223) correlation coefficient of the whole meta-analysis (Figure 2 and Figure 3). In non-diabetic subject studies, however, no study used the ECLIA method for the determination of vitamin D and the regression analysis showed no effect of method of determination of vitamin D (*R*^2^ = 0.00) (Figure 9) on the correlation coefficient.

As the sunshine vitamin, vitamin D is also expected to be effected by the latitude, and we hypothesized an effect of latitude on the correlation between vitamin D status and hyperglycemia. We also performed a meta-regression for the moderator latitude. Figure 5 shows the *R*^2^ graphics for the effect of “latitude” on the between study variability. The total variance (Tau-squared = 0.014) in the true effects was found to be 0.14% (Figure 4 and Figure 5). No variance in the effect size was observed due to latitude (*R*^2^ = 0.00) in both diabetic (Figure 5) and non-diabetic subject studies (Figure 9).

The high level of heterogeneity in the studies is most likely due to the differences in the dietary contents of vitamin D of the participants. The natural vitamin D sources are highly variable in their vitamin D content and people from different ethnicities prefer different foods depending on their traditions, culture and climate. Moreover, some participants may have vitamin D supplementations at individual level. This could be the key source of bias when pooling all 71 studies in this meta-analysis. From present study, we found that the overall heterogeneity was too high in the diabetic subject meta-analysis (*I*^2^ = 95.932%) (Figure 2) compared to the non-diabetic subject meta-analysis (*I*^2^ = 55.463%) (Figure 8). Although it may vary with the moderator “The method of determination of vitamin D”, which has not been used by the non-diabetic subject group, when we conducted the meta-analysis without those four studies (37, 38, 67, 84) using ECLIA method for the determination of vitamin D, the heterogeneity was reduced only by 1% (*I*^2^ = 94.757%). It shows that the other factors may contribute to the increase in heterogeneity in this group of study like the duration of diabetes and the complexity of the disease. For example, the diabetic patients may develop diabetic retinopathy and diabetic neuropathy and the patients of included studies may be at different stages of complexity of the disease. This heterogeneity could possibly be the result of the age group, as type 2 diabetic patients are normally of elderly age. Moreover, it is common in diabetic patients to have higher BMI compared to the non-diabetics and, as vitamin D is a fat soluble vitamin, it is trapped in adipose tissue, and very low quantities are available for circulation in the blood. The results of current meta-analysis should be interpreted critically because of different designs and methodologies adopted by these observational studies and due to the other sources of heterogeneity mentioned above.

There was an overall inverse association of vitamin D status and fasting plasma glucose in the non-diabetic population included in this meta-analysis except for a study population from United Arab Emirates [90] and an excluded study [32] (study was excluded because analysis was done in mixed population having diabetic and non-diabetic subjects) on non-Hispanic Blacks from the United States showed a positive correlation. Although this study did not have an impact on the overall statistics, it occupies a distinct position on the positive side of the forest plot showing an opposite trend compared to all other studies. The study from the United States [32] was carried out purely on Blacks who showed positive correlations, and the other study that showed positive correlations was from UAE, the inhabitants of which also migrated from Africa many years ago and have relatively darker skin shades compared to other Asians. We conclude from these results that, as the people from Africa have darker skin tones, and from years of fighting against sunlight for the production of vitamin D, they may have adapted some compensatory mechanisms to avoid the effect of vitamin D on the glucose metabolism.

The higher prevalence of hypovitaminosis D could be attributed to several factors. Mostly people do not take vitamin D fortified food and do not take vitamin D supplements, so they depend solely on UV-B rays coming from sun for their vitamin D production, therefore there is a big seasonal and climatic effect on the deficiency of circulatory vitamin D [20]. In regions where sunlight is scarce and UV rays are insufficient for the proper photosynthesis of vitamin D, the occurrence of vitamin D deficiency/insufficiency has been seen [8]. It is evident that vitamin D deficiency is prevalent all over Europe [91,92]. Vitamin D is deficient even in those countries having sufficient sunlight and day lengths [93,94,95,96]. Elderly people are especially low in their vitamin D status as their skin cannot photosynthesize vitamin D properly [97].

The link between vitamin D deficiency and the onset of type 2 diabetes could be related to genetics. It is evident that genetic factors can play a very important role in the impairment of glucose metabolism [98]. The role of genetic involvement in the onset of type 1 diabetes is well established but only few genes have been discovered to be associated with type 2 diabetes [99].

Deficiency of vitamin D is already found to be involved in the methylation of many genes [100] and also beta cells have the receptors for vitamin D [101,102], as the action of vitamin D is mediated by its receptors, it conforms the role of vitamin D in the functions of the beta cells. Studies have shown that glucose induced insulin secretion is impaired by knocking out vitamin D receptor and by inducing vitamin D deficiency [103,104,105]. The expression of insulin receptor and insulin induced uptake of glucose is stimulated by vitamin D [106,107]; these studies are supported by the facts that vitamin D receptors are present on the beta cells [108] and skeletal muscles [101,109], and vitamin D deficiency can cause insulin resistance [110] and type 2 diabetes [25]. The marker of insulin resistance has also been shown to have independent inverse relationship with vitamin D according to the previous studies. Vitamin D has also been observed to be an independent predictor of beta cell function [22]. However, many interventional studies showed no effect of vitamin D on glycaemia or insulin resistance, but it is difficult to draw conclusions from those short interventional studies, as long term deficiency cannot be justified by short term supplementation of vitamin D. In addition, many of these studies registered smaller population size and administered different formulations and doses of vitamin D. Moreover, vitamin D has low bioavailability when it is administered orally [111] and most of these interventional studies administered vitamin D orally.

Vitamin D receptor polymorphism is also expected to be associated with insulin secretion, causing reduced insulin secretion because of reduced binding affinities of vitamin D to the polymorphic receptors. Previous studies have shown that the treatment of vitamin D to isolated islets and beta cells can increase insulin secretion [101]. Diabetes is a disease which progresses to complexities easily. Previous studies have shown that vitamin D deficiency is also related with diabetic retinopathy [110] and diabetic neuropathy [39]. The *Bsm1* gene polymorphism of vitamin D receptor is known to be related with diabetic retinopathy. Hong and associates in the year 2015 found that the B allele of this gene is related with low risk of diabetic retinopathy [4]. The correction of vitamin D deficiency, especially in type 2 diabetes patients may help to prevent the progression of the disease towards complexities.

Many mechanisms might be involved in the action of vitamin D on the glucose metabolism and homeostasis, thus more detailed research is needed to uncover relationship of vitamin D with glucose metabolism. Long term randomized control trials with precise doses and formulation of vitamin D or with means of administrations other than oral route (which is thought to give low bioavailability of vitamin D) can be tried in the future.

We collected all available observational studies through systematic search strategy and we assessed the strength and quality of the evidence by using GRADE (Grading of recommendations assessment, Development and Evaluation), which is the strength of our study. The 95% Confidence intervals were not too wide, which shows the clinical importance of vitamin D for type 2 diabetes. Even though observational studies include many participants, the risk of residual confounding cannot be ruled out, which is the limitation of all observational studies compared to randomized control trials. The selection of participants in the observational studies may be the cause of confounding in this meta-analysis, as elderly age groups have low rates of photosynthesis of vitamin D from sunlight. Moreover, it is difficult to randomize and blind observational studies. These factors may contribute to heterogeneity and confounding. Considering both the strengths and weaknesses, the evidence was assessed as a moderate quality.

## 5. Conclusions

Our systematic review and meta-analysis of observational studies support an inverse association between hypovitaminosis D and type 2 diabetes. There is risk of residual confounding in the observational studies and the inconsistency between the studies increases the uncertainty in the causal effect compared to randomized control trials; in the meta-analysis of observational studies, however, we got a clear signal for the harm of hypovitaminosis D for the prevalence and incidence of type 2 diabetes, and, since the deficiency of vitamin D is currently one of the most frequent conditions globally, we suggest future large scale randomized control trials of administration of vitamin D in healthy subjects and patient populations should be performed, which would likely to have impacts on the estimates. We also suggest that the randomized control trials should be done with long term administration of vitamin D, as long-term deficiency of this vitamin cannot be justified by its short term administration. The increased inverse correlation between vitamin D status and glycemic level in diabetic (*r* = −0.223, 95% CI = −0.184 to −0.261, *p* = 0.000) versus non-diabetic (*r* = −0.073, 95% CI = −0.052 to −0.093, *p* = 0.000) subjects may suggest vitamin D as a marker of glucose homeostasis.
